# Agroecology Is Affecting Village Chicken Producers' Breeding Objective in Ethiopia

**DOI:** 10.1155/2022/9492912

**Published:** 2022-01-13

**Authors:** Minyahel Tilahun, Mosa Mitiku, Wondossen Ayalew

**Affiliations:** College of Agriculture and Natural Resources, Wolkite University, Wolkite, Ethiopia

## Abstract

This study assessed factors that determine village chicken producers' trait preferences in different agroecologies of Ethiopia. Three hundred and eighty village chicken producers were sampled for individual interviews. Data were analyzed using descriptive and inferential statistical techniques. Inbreeding coefficients of chicken populations in the three major agroecologies were estimated. In addition, the multivariate regression model was employed to evaluate the degree to which agroecological difference and socioeconomic and institutional factors impact village chicken producers' trait preferences. Egg and meat production for consumption and income generation were the three major village chicken production functions in the study. Plumage color and weight were ranked first for male and female chicken, respectively. Red plumage color (52.4%) was the primary choice followed by white color (24.5%). Agroecology and livestock holding (TLU) significantly (*P* < 0.05) affected farmers' preference toward economic traits, while land holding significantly (*P* < 0.05) affected reproductive traits. Distance to market significantly (*P* < 0.05) affected farmers' preference toward adaptive traits. The inbreeding coefficient of 0.25, 0.23, and 0.06 was recorded in low, mid, and highland agroecologies, respectively. The agroecological difference is affecting village chicken producers' breeding objective in Ethiopia. A higher inbreeding coefficient was observed in the low and mid agroecologies. Future breed improvement programs should give due consideration to village chicken producers' socioeconomic characteristics and agroecological differences.

## 1. Introduction

Village chicken production system is among the globally known agricultural production systems which has been sustained in a very shortfall condition and recognized as a vigorous strategy for capital build-up, poverty alleviation, malnutrition, and hunger reduction [1]. Nearly all poor and land-less households (HHs) in developing countries own poultry. They typically use indigenous genetic resources which are adapted to a specific harsh environment [2, 3]. Indigenous chicken breeds are claimed to be slow growers and poor producers of small-sized eggs [2]. The availability of diverse agroecologies, climatic conditions, and variation in chicken rearing purposes in the tropics are believed to contribute to the existing high chicken genetic resources diversity [4]. In Africa, nearly 85% of the estimated 1.3 billion chickens comprise indigenous breeds [5]. In Ethiopia, there is about 56.06 million chicken population, of which, 88.19% are indigenous chickens [6]. This chicken population produces 90% of egg and meat for many rural HHs as a major source of high-quality protein [7].

According to FAO [8], chicken management conditions are broadly classified into four distinct poultry production systems, i.e., small extensive scavenging, extensive scavenging, semi-intensive production, and small-scale intensive production based on flock size, production objectives, and level of specialization and/or technology use. In most chicken breed improvement endeavors, developing countries have been using high-yielding commercial breeds that have been developed for an intensive management system to increase the egg and meat production of native chicken [4]. The exotic chicken breed population has been showing a gradual increasing trend in developing countries [5]. In Ethiopia, even after long years of effort to improve the local chicken breeds' productivity through crossbreeding programs with decorated exotic chicken breeds, almost all the programs have been failed to become a sustainable food security option. Recurrently, the program's implementation strategies have been challenged by adopters due to several socioeconomic and environmental factors [9]. Furthermore, sustainable chicken production in ecologically, culturally, and economically diverse village settings is obscuring the underlying divergence which is a consequence of distinct local adaptations [10]. Globally, the conventional top-down transfer of skill and knowledge from researchers and policymakers to farmers and extension agents has been criticized for often being incompatible with the social, physical, economic, and environmental settings in which farmers operate.

Developing proper animal breeding programs for village conditions needs to understand the production environments, different management practices, production objectives, and trait preferences of producers [11]. Despite their importance, various factors such as the transition to new production systems, indiscriminate crossbreeding, and overlooking environmental and socioeconomic factors have been failing the expected success of the breeding programs [9]. Particularly, considering adaptive, reproductive, and economic traits is very crucial for chicken breed conservation and production improvement for the development of sustainable breeding strategies [2].

Even if Ethiopia has diverse agroecological zones paired with a huge number of plant and animal species, for a long, there have been few studies that characterized the existing system and available local chickens' populations [2, 12–15]. Lately, indigenous chicken genetic improvement program has been initiated aiming for livelihood improvement and genetic diversity conservation [2, 16]. Although adaptive attributes and genetic merits of indigenous chickens are strongly correlated with the level of farmers' preference traits, only very few studies tried to show their relationship [2, 9]. In addition, economic attributes complement or stimulate further work on the economic valuation of the morphological traits [17]. Even in the absence of modern measurement, relative weights of the desired trait could be used to suggest an alternative breeding plan [18]. Farmers in traditional systems rate trait categories based on economic grounds which could be converted into economic weights [19].

The Gurage zone is among a few areas of Ethiopia where clear altitudinal and agroecological differences are exhibited within a very short distance [20]. It is also among a few areas of the country where many years of planned and unplanned chicken breeding programs have been implemented. A possible intervention to improve village poultry production is to target and improve indigenous breeds based on the needs and preferences of smallholder farmers [2]. However, information regarding farmers' trait preference determinant factors considering agroecological classification is still very few. Understanding this gap can help us to identify foregoing farmers' views and preference toward conception of new trait due to frequent interventions. Therefore, the objective of this study was to assess and prioritize village chicken producers' trait preferences and identify factors that determine such preference toward chicken production at different agroecologies.

## 2. Materials and Methods

### 2.1. Description of the Study Area

**Figure 1:**
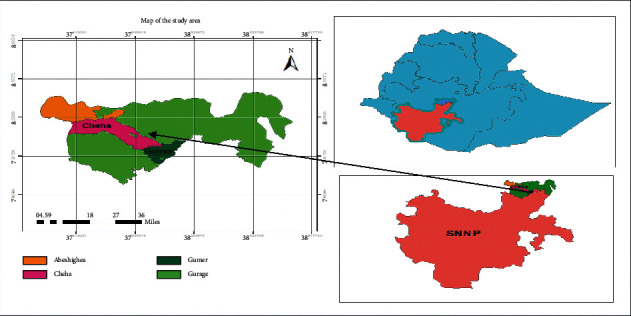
Map of the study area.

Gurage Zone is found 158 km from the capital city of Ethiopia, Addis Ababa. The major agricultural activity is subsistence farming. The human population of the zone was 1.83 million [20]. The study area is geographically located between 8° 00' 18.9” to 8° 15' 28.53” North and 37° 35' 46.48” to 38° 03' 59.59” East. The zone hosts all the three major agroecologies, i.e., lowland, midland, and highland. The average rainfall of the zone ranges between 801 and 1400 mm, and the average temperature of the study zone ranges between 7.5 and 25°C (Figure 1). The main food crops grown in the area are enset, maize, teff, beans, bananas, barley, potato, and vegetables such as pepper, tomatoes, onions, and cabbages. The main cash crops are coffee and khat. Around 1.8 million livestock are found within the zone. There are around 2.88 million chickens found in the zone. In this study, the first district, i.e., Gumer, represents the highland ecology and receives annual rainfall ranging from 1001 to 1400 mm, and the annual temperature is ranging from 12.6 to 22.5°C. The second district, i.e., Cheha, represents the midland ecology and receives annual rainfall ranging from 801 to 1400 mm and the annual temperature is ranging from 17.6 to 25°C. The third district, i.e., Abeshghea, represents the lowland ecology and receives annual rainfall ranging from 801 to 1400 mm and the annual temperature is ranging from 17.6 to 30°C [20].

### 2.2. Data Type and Source

Sample respondents were randomly selected from the list of HHs who own chicken provided by the agricultural development offices of the selected districts. Both qualitative and quantitative data types were used. The quantitative data types were used to estimate the socioeconomic characteristics of respondents and the inbreeding coefficient of chicken populations in the study area. Qualitative data types were used to assess respondents' trait preferences, the function of village chicken production, attributes used by farmers to select chickens, and different management systems used by village chicken producers. Those traits can award them the survival advantage and enable them to reproduce with relatively high rates and pass on the successful genotypes to subsequent generations. Adaptive traits such as ability to escape from predators, disease, and stress tolerance and required management level were considered for this study. Economic traits such as scavenging behavior, hatchability of eggs, taste of egg, yolk color of egg, and egg and meat production capacity were considered for this study. Environmental, physiological, and morphological changes may bring constant phenotype change throughout the life of an individual. Even if more than four morphological traits were identified in the study, only four of the frequently mentioned traits were given emphasis. Evaluated village chicken traits of this study were presented in local language called “*Guragina*.”

### 2.3. Research Design and Sampling Technique

The study sites were selected by considering the differences in the agroecologies and respondents' socioeconomic characteristics. Subsequently, three districts, i.e., Abeshghea, Cheha, and Gumer, were selected from the lowland (LL), midland (ML) and highland (HL) agroecologies, respectively.

### 2.4. Sample Size Determination

After selecting the study districts, sample HHs' size was determined based on the formula by [21] as follows:

(1) $$\begin{array}{c} n=\frac{N}{1+N{\left(e\right)}^{2}},\\ n=\frac{69798}{1+69798{\left(0.05\right)}^{2}}=380, \end{array}$$

where *n* is the sample size, *N* is the total population, and 1 is the constant *e* = margin of error (*e* = 0.05). Eventually, a total of 380 HH samples were determined. The majority of the chicken populations in Ethiopia is found in the midland agroecology, and the two extremes, i.e., highland and lowland agroecologies, have comparably low chicken populations. Environmental suitability, physiological adaptability, and availability of preferred feed resources for chicken made midland agroecology more suitable for the chicken population. Therefore, midland agroecology (*Cheha*) took the highest proportion (50%) of the sample size and the rest 50% of the sample was equally shared to high (*Gumer*) and low (*Abeshghea)* agroecologies. Two kebeles were selected from each of the three districts for HH data collection purposes. Kebele represents the smallest administration level in Ethiopia. They were selected based on their history of participation in village chicken breed improvement programs in consultation with the district's agricultural offices. Finally, 190 HHs from the midland and 95 HHs from the highland and lowland agroecologies each were in the HH survey. HHs were selected randomly from the list of village chicken-producing HHs collected from the selected kebeles that own more than one chicken and have chicken rearing experience.

### 2.5. Data Collection and Analysis

Individual interview using semistructured questionnaire was employed. In addition, focus group discussion (FGD) regarding trait preference, selection decisions, market value, and personal observation was used for triangulation purposes. Three FGDs, i.e., one from each agroecology, were conducted. Each group was designed to incorporate 7–10 participants. The discussion participated elders, extension agents, merchants, farmers, and experts in the field.

The individual interview was conducted with the help of two enumerators who have more than 5 years of work experience in the study area and speak *Guragina*. It was designed to collect two kinds of data. The first covered respondents' socioeconomic information and livestock and chicken holdings. The second kind focused on chicken management, production objectives, population structure, breed choice, market traits' preference, and farmers' selection practices. Subsequently, the results of the individual interviews were summarized according to (1) relative importance of different functions of chickens, (2) attribute used by farmers to select chicken, (3) feeding, housing, and health management systems, and (4) effective chicken population structure.

The consensus regarding village chickens' production function and traits' preference in the three agroecologies was calculated based on the collected response from village chicken producers' preference. The score given for the different functions of village chicken production and major attributes used as selection criteria was collected and used to calculate their index and rank. The index was calculated using the following formula:

(2) $$\begin{array}{c} \text{Index}=\;\frac{Rn*C1+Rn-1*C2+\cdots +R1*Cn}{\sum Rn*C1+Rn-1*C2+\cdots +R1*Cn}, \end{array}$$

where *Rn* is the value given for the least ranked level (for example, if the least rank is 6th, then *Rn* = 6, *Rn* − 1 = 5,…, *R*1 = 1); *Cn* is the count of the least ranked level (in the count of the 1st rank = *C*1). The opposite matching for *R* and *C* values can be presented as follows:

*R*1 for *Rn*, *R*_2_ for *R*_*n*-1_…, *R*_n_ for *R*_1_ and *C*_1_ for *C*_*n*_, C_2_ for *C*_*n*-1_,…, *C*_*n*_ for *C*_1_.

The effective population size of the three agroecologies was determined by the following formula [22]:

(3) $$\begin{array}{c} Ne=\frac{\left(4*Nm*Nf\right)}{\left(Nm+Nf\right)}, \end{array}$$

and the increase in inbreeding per generation as

(4) $$\begin{array}{c} \Delta F=\frac{1}{\left(2Ne\right)}, \end{array}$$

where *Ne* is the effective population size, *Nm* is the number of breeding males, *Nf* is the number of breeding females, and Δ*F* is the inbreeding coefficient.

After profound scrutiny and debugging, the collected data were analyzed using SPSS statistical software version 21 [23]. Data were assumed to be statistically significant at *P* < 0.05.

### 2.6. Variables' Description

The major chicken production objective traits, i.e., economic, adaptive, and reproductive, were considered for the regression analysis estimated in this study. Adaptive traits are given major emphasis based on the degree of dryness and harshness of the production environment. Both reproductive and economic traits get special consideration in the mid and high agroecologies with slight variation in their degree of implementation due to factors such as distance to the nearest market, extension service delivery, landholding size, and farmers' education level [17].

Agroecology of land (AGRECO), gender (GEN), distance to the nearest market (DISTMKT), and veterinary service (VETSER) are dummy variables represented by 0 and 1. These factors have a positive effect on farmers' trait preferences. On the contrary, age of HH head (AGE), education level (EDU), chicken population (CHPO), livestock holding in TLU (LHO), and land size (LAND) are continuous variables measured in different linear measurements. As these factors increase/decrease, they can positively or negatively affect the dependent variables.

### 2.7. Model Specification

Multivariate regression is a method used to measure the degree to which more than one independent variable (predictors) and more than one dependent variable (responses) are linearly related. The method is broadly used to predict the behavior of the response variables associated with changes in the predictor variables, once a desired degree of relation has been established [24]. In this study, this model was employed to measure the degree to which village chicken producers' preference toward their breeding objective was affected by different socioeconomic factors.

The multivariate regression model relates more than one predictor and more than one response. Let *Y* be the *n* × *p* response matrix; let *X* be an *n* × (*q* + 1) matrix such that all entries of the first column are 1's and *q* predictors. Let *β* be a (*q* + 1) × *p* matrix of fixed parameters; let *ε* be an *n* × *p* matrix such that *ε* ∼*N* (0, Σ) (multivariate normally distributed with covariance matrix Σ). The model is as follows:

(5) $$\begin{array}{c} Y=x\beta +\varepsilon \\ \left(\begin{array}{cccc} y_{11} & y_{12} & \ldots & y_{1p}\\ y_{21} & y_{22} & \ldots & y_{2p}\\ y_{31} & y_{32} & \ldots & y_{3p}\\ \vdots & \vdots & \ddots & \vdots \\ y_{n1} & y_{n2} & \ldots & y_{np} \end{array}\right)\\ =\left(\begin{array}{ccccc} 1 & x_{11} & x_{21} & \ldots & x_{1q}\\ 1 & x_{21} & x_{22} & \ldots & x_{2q}\\ 1 & x_{31} & x_{32} & \ldots & x_{3q}\\ \vdots & \vdots & \vdots & \ddots & \vdots \\ 1 & x_{n1} & x_{n2} & \ldots & x_{nq} \end{array}\right)\left(\begin{array}{cccc} \beta_{01} & \beta_{02} & \ldots & \beta_{0p}\\ \beta_{11} & \beta_{12} & \ldots & \beta_{1p}\\ \beta_{21} & \beta_{22} & \ldots & \beta_{2p}\\ \vdots & \vdots & \ddots & \vdots \\ \beta_{q1} & \beta_{q2} & \ldots & \beta_{qp} \end{array}\right)+\left(\begin{array}{cccc} \varepsilon_{11} & \varepsilon_{21} & \ldots & \varepsilon_{1p}\\ \varepsilon_{21} & \varepsilon_{22} & \ldots & \varepsilon_{2p}\\ \varepsilon_{31} & \varepsilon_{32} & \ldots & \varepsilon_{3p}\\ \vdots & \vdots & \ddots & \vdots \\ \varepsilon_{n1} & \varepsilon_{n2} & \ldots & \varepsilon_{np} \end{array}\right). \end{array}$$

## 3. Results

### 3.1. Respondent Households Characteristics

The ecological characteristic of the study area is presented in Table 1. The study areas that represent the three agroecologies varied in terms of human population size, average family size, the total number of chickens, and average flock size. Among the ecologies, the HL had the highest average family size (4.9) compared to the rest districts. The total number of chickens found in the ML (249, 827) was higher than the rest two agroecologies. The average flock size of the ML was ranked first (8.7) followed by the HL (3.9) and the LL (3.1), respectively.

### 3.2. Socioeconomic Characteristics of Village Chicken Producers in Central Ethiopia

The socioeconomic characteristic of respondents of this study is presented in Table 2. The average age of the respondents of this study was 46.25 years old. Of the total respondents, the majority of the respondents that participated in this study were male-headed HHs (82.37%). The proportion of respondents that did not have formal education was 21%, and among the three agroecologies, a higher proportion of illiteracy was recorded in the HL agroecology (26%). The average family size of the study population was 7.1. Among the three agroecologies, a higher average family size (7.8) was recorded in the HL agroecology.

### 3.3. Functions of Village Chicken Production

The relative importance of different functions of village chicken production is presented in Table 3. In all of the agroecologies, egg for home consumption was ranked first among the different functions of chicken production in all agroecologies. Meat for home consumption was ranked second in the HL and ML agroecologies. However, in the LL agroecology, chicken for cultural use took the second rank. Chicken production for income generation was ranked third in all agroecologies. Chicken production for religious purposes took the last rank in all the agroecologies.

### 3.4. Major Village Chicken Management Practices

The major village chicken management practices of the study are presented in Table 4. The major management practices identified in the study were feeding, housing, and health management. Concerning the feeding practices, scavenging + supplementation was practiced by 52.9% of the respondents followed by sole scavenging (43.7%). Concerning the housing of chickens, almost half (46.5%) of the respondents kept chickens inside the family house to perch followed by keeping chickens separately from other livestock (41.1%). Regarding the health management practices, the majority (92.4%) of the respondent HHs were using traditional health management systems followed by modern and mixed health management systems.

### 3.5. Village Chicken Producers' Preferences toward the Selection of Chicken

Table 5 presents major village chicken producers' preferences toward the selection of chickens. All adaptive traits, i.e., ability to escape from predators, disease and stress tolerance, and scavenging vigor showed significant importance in all agroecologies. Among the attributes, low preference was observed toward production/economic traits in all agroecologies. The trait of meat production was preferred by only 26% of respondents of this study. A low proportion of respondents was observed on farmers' trait preference toward egg hatchability in all agroecologies, i.e., HL (47.37), ML (50.5), and LL (66.3). Required management levels showed a lower proportion in both LL (42.1) and HL (47.3) agroecologies.

### 3.6. Farmers' Selection Practices

Prompting factors toward the price of live chickens market and farmers' preferences for specific traits in plumage colors and comb types are presented in Tables 6 and 7. Farmers from the HL agroecology gave the highest rank to plumage color whereas live weight ranked first in both the ML and LL agroecologies. A difference in trait preference was exhibited between male and female sex categories. Female chicken plumage color was ranked first in all agroecologies followed by breed and comb type, respectively. In addition, the weight of chickens ranked least in all of the agroecologies except the HL. Significant variation in the ranks of the preferred trait was exhibited among the different agroecologies. Although each of the identified trait categories consisted of different components, farmers described only two of the four trait categories used as selection criteria, i.e., plumage color and comb type. White (*Guwad*) and red (*Bisha*) colors were identified as the two important plumage colors in the study area. Red color (52.4%) was the most favored plumage color in all of the agroecologies, whereas white plumage color (24.5%) took the second proportion as compared to the rest color choices. Similarly, almost all respondents (94%) of the study areas recognized two comb types, i.e., “*Tiletiye*” and “*Difdif*.” “*Tiletiye*” comb type represents Single and “*Difdif*” actually comprised all comb types other than “Single” (i.e., rose, pea, walnut, and duplex combs). “*Difdif*” was the favored comb type (93.2%) in all of the agroecologies. Other traits, i.e., weight and breed, did not have established characteristics that were readily identified by the community. Rather, the community just made a comparison among the existing trait by setting a reference trait.

### 3.7. Multivariate Regression Model Output

Table 8 presents factors that determine the preference of village chicken-producing farmers. The effect of different socioeconomic and institutional factors on village chicken producers' trait preferences was determined using the multivariate regression model. A significant (*P* < 0.05) difference was observed between the difference in agroecology and farmers' trait preferences toward economic traits. Farmer's total livestock holding (TLU) was significantly (*P* < 0.05) related to economic traits. A significant difference (*P* < 0.05) was observed between farmers' landholding size and their choice of reproductive traits. Moreover, a significant difference (*P* < 0.05) was also observed between farmers' village distance from the local market in relation to their choice toward adaptive traits.

### 3.8. Inbreeding Coefficient of Village Chicken Population of the Study Area

Possession of breeding males, effective population size, and the level of inbreeding of village chicken population of the study area are presented in Table 9. Highland agroecology chicken population showed a very low inbreeding coefficient as compared to other agroecologies. Among the agroecologies, a higher inbreeding coefficient (0.25) was observed in the LL agroecology, a higher breeding male was recorded in the HL agroecology (36.8%), and a lower proportion (20%) of breeding males was recorded in the LL agroecology. The highest and the lowest inbreeding coefficient was recorded in the LL (0.25) and HL (0.06) agroecologies, respectively.

## 4. Discussion

The village chicken production system is characterized by extensive scavenging, no immunization programs, high prevalence of disease and predators, and uncontrolled natural mating and hatching of eggs using broody hens [9]. Many African countries produce chicken through this system [25–27]. Similarly, the majority of Ethiopian chicken producers have no particular breeding objective and are also known for their extensive way of production and fragile management. This study finding indicates that egg production for consumption is the primary function of village chicken production. In line with this study finding, Dana et al. [9] and Bettridge et al. [10] reported that egg and meat production is the principal breeding objective of village chicken production. Chicken functions as a source of income and security [8] and cultural and religious roles are also another function of village chicken production in different parts of Ethiopia [28, 29].

In the last few decades, intensive and progressive breed improvement programs have been implemented in Ethiopia. In addition to the expected production improvement of the programs, they also bring numerous new morphological changes to the already characterized local chicken breeds. One of the deep-seated reasons for this unplanned change might be the lack of particular breed improvement program owners and overlooking recommended suitable chicken breeds for specific agroecology. Due to the hysterical chicken improvement programs of the country, it is easy to observe new and spontaneous chicken traits principally in rural and urban markets. This might gradually shape the acceptance of new morphological traits in rural chicken markets [11]. This study finding reveals that only 53% of the total village chicken producers (210) provided supplementary feed to their chicken. In contrast to this finding, almost all village chicken producers in Ghana and Mozambique offered supplementary feeds to their chicken [25–27]. Knowledge and skill of proper feeding are acquired through the provision of continuous and problem-based technical training and workshops to farmers which are directly associated with production improvement [30]. The study finding also indicates that only 52% of village chicken producers kept and perched their chickens at night inside the living room. In contrast, African countries like Mozambique village chicken producers experienced the construction of separate chicken shelters which bring significant production and productivity improvement in chicken [27].

Adaptability to the environment is generally described in terms of different traits enabling them to survive, reproduce, and be productive in resource-limited and harsh production conditions [31]. Earlier studies [32, 33] on the adoption of new chicken breeds to Ethiopia indicate that morphological traits such as plumage color and comb types have been given significant emphasis in the process of selecting chicken. These traits determine the adoption efficiency of imported chicken breeds besides other quantitative traits. On the contrary, village chicken producers of many west, central, and east Africa and Asia countries consider egg production as the major breeding stock selection criterion followed by mothering ability and body weight [34]. Village chicken producers' preference toward the selection of traits across agroecology is in line with the submission of Mengesha and Tsega [35]. The exhibited variation across the agroecologies could be associated with the breed improvement programs recorded benefits or failures at the country level. Consequently, it is worthwhile to filter out the traits preference of farmers from different agroecologies toward their breeding objectives [11].

Studies by Dana et al. [9], Chebo and Dana [33], and Jiang [36] indicate that plumage color is highly preferred by market participants followed by chicken weight and comb type in both Ethiopia and China, respectively. Findings from the undertaken FGDs indicate that village chicken producers' plumage color preference is directly associated with the chicken's ability to survive within the defined environment. On the contrary, feather distribution has equal importance with plumage color [37]. A slight trait preference difference regarding morphological trait differences between male and female chickens was observed. This is in contrast with [9] in different parts of Ethiopia. The introduction of several unrepressed exotic chicken breeds to the central part of Ethiopia might be the reason for the exhibited village chicken producers traits' preference difference. In addition, the majority of previous studies only considered characterized local chicken breeds by overlooking the impacts of unplanned crossbreeding and market influence on the available chicken populations in different parts of Ethiopia. This study indicates that farmers' trait preferences are less affected by cocks' plumage color as compared to chicks. In line with this finding, Terefe et al. [6] indicated that farmers' preference difference was observed due to factors such as respondents' gender difference, agroecologies, and study populations.

Even if qualitative traits such as plumage color (red and white) and comb type have got huge consideration, important quantitative traits such as growth traits obtained trivial emphasis. This is supported by Moges et al. [38] that indicate chickens with red or white plumage colors combined with pea-shaped comb types have higher market prices. Similarly, farmers show stronger affection for white plumage color compared to other colors. On the contrary, there are studies that indicate the significant influence of body size and general body condition on the price of chickens [39–41]. Egg size is highly preferred by farmers to select female chicken [42]. In opposition to our finding, farmers in Zimbabwe gave no emphasis to plumage color; rather they traditionally selected chicken based on other qualitative traits such as compact [41]. In Nigeria, live weight was the most important attribute compared to other traits [43]. However, the exhibited difference in trait preference difference might arise due to the fact that separate trait assessments regarding sex, breed, and production objectives have not been done. This trait category has been described similarly and attributed a comparable level of importance in other species of livestock produced by village farmers [44].

Though this study indicates the direct association between farmers' trait preferences and adaptive traits, the selection practices are also highly influenced by market price differentials. For instance, very important economic traits such as egg production for market supply have not been given due emphasis by village chicken producers. The preference traits in both male and female chickens have been majorly dependable on those traits preferred by local market participants because chickens are also means of income in times farmers are bankrupt. The laying performance of parent stock chickens can indicate the best female and male offspring which can be used for the replacement of next-generation egg producers. Lack of instant information regarding production performance is the basic problem that challenges farmers to trust production performance information. Except for morphometric traits, important traits that indicate adaptability, egg production, and reproductive performance are not properly employed in the majority of village chicken producers' selection criteria [41–43].

A significant relationship (*P* < 0.05) between agroecology and the economic trait was observed. The proportional difference in chicken products sold among the three agroecologies might be associated with the observed significant difference. Moreover, the observed significant (*P* < 0.05) relationship between total livestock holding (TLU) and economic traits can be an indicator for chicken production and the market experience of the farmers. Landholding size also had a significant relationship (*P* < 0.05) with farmers' preference toward reproductive traits. Respondent home distance to market also had a significant (*P* < 0.05) relationship with farmers' preference toward adaptive traits. In areas where markets are far from villages, supplying chicken products to market becomes very difficult, and it makes farmers less beneficial from the production due to extra investment on transportation and accommodation. Consequently, village chicken producers' prefer more adaptive traits than economic traits. This finding is supported by [45] which indicates some adaptation strategies suggested by the Africa-wide results are not appropriate for a specific agroecology. For instance, variation in the trait preference of farmers from different agroecologies can easily be observed in relation to adaptation to the production environment (ability to escape from predators; scavenging behavior) which is the most important attribute in all agroecologies coupled with a proper management system. According to Niggol et al. [46], the choice of which species to select is slightly more difficult in breed improvement programs because there are more choices in their surroundings.

The number of chickens in high elevation regions increases but falls in low elevation regions [46]. The effective population size of this study ranges from 1.98 to 8.1 in LL and HL agroecologies, respectively, of which, the average breeding male chicken number is very small in all the agroecologies. The average chicken population size (4.75) of the study area is higher than the country average (2.3). The population is very low as compared to neighboring country Kenya (31.8) in 2019 [34]. Although the chicken population of the study area shows an increasing trend, World Bank predicted chicken population size decline by the year 2020 depending on the climate scenarios [46]. The higher inbreeding coefficient in the LL and ML agroecologies as compared to the HL agroecology might be due to market demand on cocks and cockerels selling. In addition, the very small average chicken flock size can confirm the drastic drop in the total population of the country since the past decade [9]. From the researchers' personal observation, the local chicken market is an important source of breeding males that might contribute toward reducing further inbreeding. Inbreeding can possibly be very high in the scavenging chickens as the breeding stock is rarely replaced by flocks from outside of the HH. Melese [45] reports that the occurrence of inbreeding in scavenging flocks can lead to depression in production potential, hatchability, and survivability of the flocks. In the long run, if no measure is taken to decline the inbreeding in the agroecologies with a higher inbreeding coefficient, the production potential of the population in that agroecologies will decline and be unable to adapt easily. Data collection biases can erroneously arbitrate effective population size as well as the rate of inbreeding coefficient due to uncontrolled natural mating and the absence of breeding males in the majority of village chicken-producing areas.

## 5. Conclusion

1. This study concludes that egg production for consumption was the major function of village chicken production followed by meat production and income generation, respectively.
2. Variability in agroecology and frequent introduction of new chicken breeds had brought frequent changes in breeding objectives in village chicken production. This can be an absolute witness to the importance of imperative and controlled indigenous chicken genetic improvement programs at the farmer's level.
3. Female chicken selection was primarily arbitrated by plumage color followed by breed and comb type whereas the male chicken selection was governed by weight followed by plumage color and comb type, respectively.
4. Adaptive traits were obtained as the highest village chicken producers' traits preference compared to other major traits (productive and reproductive) in all the agroecologies. A lower inbreeding coefficient was recorded in the highland as compared to lowland and midland agroecology. Farmers' preference toward traits other than economic traits was determining the coefficient of the existing chicken variability, particularly in the highland agroecology.

Therefore, in future chicken breeding strategies development, farmers' definitions regarding trait categories rating should have to be given due emphasis. Designed policies that facilitate a uniform approach toward adaptation to different agroecologies should be avoided and customized to host variability in the implementation of new breed adaptation to an area. This can further help the development of productive chicken breeds that can sustainably produce, survive, and reproduce under different production environments.

## Figures and Tables

**Table 1:** Ecological characteristics and human and chicken flock size of study districts (mean ± SD).

Ecology	Agroecologies			Total
	HL	ML	LL	
Human population	109, 888	120, 597	83, 484	104656.33 ± 15596.4
Average family size	4.9	4.2	4.5	4.53 ± 0.286
Number of household	22533	28,713	18,552	23,266 ± 4180.46
Total number of chickens	89379	249,827	58,272	132,493 ± 83934
Average flock size	3.9	8.7	3.1	5.2 ± 2.47

The three districts, i.e., Gumer, Cheha, and Abeshigea, represent HL (highland), ML (midland), LL (lowland), respectively; *N* is the total number of respondents.

**Table 2:** Socioeconomic characteristics of village chicken producers (*n* (HL : LL) = 95; ML = 190; *N* = 380).

Description of households		Agroecologies (%)			Total
		HL	ML	LL	
Age (mean)		52	48	39	46.25 ± 5.43
Sex of household head	Male	70 (73.7)	162 (85.2)	81 (85.2)	82.37
	Female	25 (26.3)	28 (14.7)	14 (14.8)	17.63
Educational level	Uneducated	25 (26.3)	40 (21)	15 (15.8)	21.05
	Literate	70 (73.7)	150 (79)	80 (84.2)	78.95
Family size (mean)		7.8	7.1	6.5	7.1 ± 0.53
Livestock owned	Cattle	4.2	6.8	4.3	5.1 ± 1.446
	Sheep	1	1.4	0.2	0.87 ± 0.5
	Goat	0.74	2.9	0.8	1.5 ± 1
	Chicken	8.7	3.15	3.1	4.5 ± 2.67
Land owned size (hectares)		1.6	1.4	1.6	1.53 ± 0.094

HL: highland; ML: midland; LL: lowland; *N*: total number of respondents.

**Table 3:** Relative importance of different functions of chickens (*n* (HL : LL) = 95; ML = 190; *N* = 380).

Functions of chicken	Agroecologies						Total	
	HL		ML		LL			
	*n* (index)	Rank	*n* (index)	Rank	*n* (index)	Rank	*N* (index)	Rank
Egg for consumption	427 (0.3)	1	812 (0.28)	1	391 (0.27)	1	1630 (0.29)	1
Meat for consumption	305 (0.21)	2	586 (0.21)	2	281 (0.197)	4	1172 (0.21)	2
Cultural	246 (0.17)	4	564 (0.197)	4	306 (0.21)	2	1116 (0.196)	4
Income generation	285 (0.2)	3	570 (0.1990	3	285 (0.2)	3	1140 (0.199)	3
Religion	165 (0.11)	5	324 (0.11)	5	162 (0.11)	5	648 (0.11)	5

HL: highland; ML: midland; LL: lowland; *N*: number of respondents.

**Table 4:** Major village chicken production management practices of the study area (n (HL : LL) = 95; ML = 190; *N* = 380).

Management practices	Agroecologies *n* (%)			Total *N* (%)
	HL	ML	LL	
*Feeding*				
Scavenging	35 (36.8)	86 (45.3)	45 (47.4)	166 (43.7)
Scavenging + supplement	55 (57.9)	98 (51.6)	48 (50.5)	201 (52.9)
Confined, complete ration	5 (5.2)	6 (3.2)	2 (2.1)	13 (3.4)

*Housing*				
Inside the family house	54 (56.8)	84 (44.2)	39 (41.1)	177 (46.5)
Separate shelter but with other livestock	32 (33.7)	84 (44.2)	40 (42.1)	156 (41.1)
Separate shelter	9 (9.5)	22 (11.6)	16 (16.8)	47 (12.4)

*Health*				
Traditional	84 (88.4)	179 (94.2)	88 (92.6)	351 (92.4)
Modern	6 (6.3)	6 (3.2)	5 (5.2)	17 (4.5)
Mixed	5 (5.2)	5 (2.6)	2 (2.1)	12 (3.2)

HL: highland; ML: midland; LL: lowland; *N*: number of respondents.

**Table 5:** Major attributes used by farmers to select chicken (n (HL : LL) = 95; ML = 190; *N* = 380).

Major attributes		Agroecologies *n* (%)			Total *N* (%)
		HL	ML	LL	
Ability to escape from predators		90 (94.7)	173 (91.1)	89 (93.7)	352 (92.6)
Disease and stress tolerance		81 (85.2)	181 (95.3)	89 (93.7)	351 (92.7)
Management level required		45 (47.3)	182 (95.8)	40 (42.1)	267 (70.2)
Scavenging behavior^*∗*^		92 (96.8)	187 (98.4)	95 (100)	374 (98.4)
Hatchability of eggs^*∗*^		45( 47.37)	96 (50.5)	63 (66.3)	204 (53.7)
Taste^*∗*^	Egg	89 (93.7)	184 (96.8)	85 (89.5)	358 (94.2)
	Meat	93 (97.9)	182 (85.8)	88 (92.6)	363 (95.5)
Yolk color of egg^*∗*^		93 (97.9)	187 (98.4)	93 (97.9)	373 (98.2)
Production capacity^*∗*^	Egg	48 (50.5)	96 (50.5)	39 (41.1)	183 (48.2)
	Meat	56 (58.5)	49 (25.8)	41 (43.2)	146 (38.4)

HL: highland; ML: midland; LL: lowland; *N*: number of respondents. The sign ^*∗*^represents economic traits.

**Table 6:** Prompting factors toward the price of live chickens marketed in the study area (*n* (HL : LL) = 95; ML = 190; *N* = 380).

Trait category/factor	Agroecologies						Total	
	HL		ML		LL			
	*n* (index)	Rank	*n* (index)	Rank	*n* (index)	Rank	*N* (index)	Rank
*Male*								
Plumage color	279 (0.29)	1	536 (0.28)	2	268 (0.28)	2	1083 (0.29)	2
Live weight	259 (0.27)	2	581 (0.31)	1	301 (0.32)	1	1141 (0.30)	1
Comb type	239 (0.25)	3	461 (0.24)	3	217 (0.23)	3	917 (0.24)	3
Breed	173 (0.18)	4	322 (0.17)	4	164 (0.17)	4	659 (0.17)	4

*Female*								
Plumage color	279 (0.29)	1	574 (0.30)	1	268 (0.28)	1	1121 (0.3)	1
Live weight	226 (0.24)	3	386 (0.20)	4	217 (0.23)	4	829 (0.22)	4
Comb type	250 (0.26)	2	407 (0.21)	3	241 (0.25)	2	898 (0.24)	3
Breed	195 (0.21)	4	533 (0.28)	2	224 (0.24)	3	952 (0.25)	2

HL: highland; ML: midland; LL: lowland.

**Table 7:** Farmers' preferences toward major morphological traits.

Major traits *(Guragina)*	Agroecologies *n* (%)			Total *N* (%)
	HL	ML	LL	
*Plumage color*				
White (*Guwad*)	30 (28.5)	46 (24.2)	17 (16.5)	93 (24.5)
Mixed color (*Arekot*)	11 (10.45)	25 (13.16)	16 (15.2)	52 (13.7)
Speckled (*Kekebetye*)	9 (8.55)	17 (8.9)	10 (9.5)	36 (9.5)
Red (*Bisha*)	46 (43.7)	103 (54.2)	50 (47.5)	199 (52.4)
Black (*Tikur*)	2 (1.9)	3 (1.6)	2 (1.9)	7 (1.8)
Any other	2 (1.9)	6 (3.2)	5 (4.75)	13 (3.4)

*Comb type*				
Single (*Tiletiye*)	5 (4.75)	15 (7.9)	3 (2.85)	23 (6.1)
Double(*Difdif*)	90 (85.5)	172 (90.5)	92 (87.4)	354 (93.2)
Any other	5 (4.75)	13 (6.8)	5 (4.75)	23 (6.1)

HL: highland; ML: midland; LL: lowland; *N*: number of respondents.

**Table 8:** Effect of determinant variables on farmers' trait preference.

Source	Dependent variable	SS	df	MS	F	Sig.
Agroecology (AGRECO)	Adaptive	0.345	2	0.172	1.022	0.361
	Economic	1.526	2	0.763	4.204	0.016 ^*∗*^
	Reproductive	0.198	2	0.099	1.461	0.234
Age (AGE)	Adaptive	0.132	3	0.044	0.261	0.853
	Economic	0.723	3	0.241	1.328	0.266
	Reproductive	0.489	3	0.163	2.398	0.068
Gender (GEN)	Adaptive	0.041	1	0.041	0.244	0.622
	Economic	0.006	1	0.006	0.034	0.854
	Reproductive	0.008	1	0.008	0.124	0.725
Education (EDU)	Adaptive	1.103	3	0.368	2.180	0.091
	Economic	0.880	3	0.293	1.617	0.186
	Reproductive	0.428	3	0.143	2.098	0.101
Chicken population (CHPO)	Adaptive	0.756	2	0.378	2.241	0.108
	Economic	0.013	2	0.006	0.035	0.965
	Reproductive	0.102	2	0.051	0.748	0.475
Livestock holding in TLU (LHO)	Adaptive	10.681	71	0.150	0.892	0.712
	Economic	17.677	71	0.249	1.372	0.040 ^*∗*^
	Reproductive	5.119	71	0.072	1.062	0.362
Land size (LAND)	Adaptive	3.498	18	0.194	1.152	0.302
	Economic	3.661	18	0.203	1.121	0.332
	Reproductive	2.208	18	0.123	1.806	0.025 ^*∗*^
Distance to market (DISTMKT)	Adaptive	1.491	2	0.746	4.422	0.013
	Economic	0.924	2	0.462	2.544	0.080
	Reproductive	0.303	2	0.152	2.233	0.109
Veterinary service (VETSER)	Adaptive	0.039	1	0.039	0.230	0.632
	Economic	0.126	1	0.126	0.696	0.405
	Reproductive	0.028	1	0.028	0.410	0.523
Error	Adaptive	44.016	261	0.169		
	Economic	47.369	261	0.181		
	Reproductive	17.725	261	0.068		
Total	Adaptive	290.000	380			
	Economic	206.000	380			
	Reproductive	29.000	380			
Corrected total	Adaptive	68.684	379			
	Economic	94.326	379			
	Reproductive	26.787	379			
(a) *R* squared = 0.359 (adjusted *R* squared = 0.069)						
(b) *R* squared = 0.498 (adjusted *R* squared = 0.271)						
(c) *R* squared = 0.338 (adjusted *R* squared = 0.039)						

df: degree of freedom; MS: mean square; SS: sum of squares;  ^*∗*^ represents *P* ≤ 0.05.

**Table 9:** Possession of breeding males, effective population size, and level of inbreeding of village chicken flock in the study area.

Agroecologies	Total number of respondents (*N*)	Not possess breeding males *N* (%)	Possess breeding males (%)	*Nm*	*Nf*	*Ne*	Δ*F*
HL	95	16(16.85)	36.8	3.22	5.5	8.1	0.06
ML	190	22 (11.6)	21.6	0.7	2.45	2.17	0.23
LL	95	23(24.2)	20	0.62	2.48	1.98	0.25

*Nm*: number of breeding males; *Nf*: number of breeding females; *Ne*: effective population size; Δ*F*: inbreeding coefficient; HL: highland; ML: midland; LL: lowland; *N*: number of respondents.

## Data Availability

The datasets used and/or analyzed during the current study are available from the corresponding author on reasonable request.

## Abbreviations

CSA: Central Statistical Agency
°C: Degree Celsius
FGD: Focus group discussion
FAO: Food and Agricultural Organization
GZFEAO: Gurage Zone Finance Economy Administrative Office
HHs: Households
HL: Highland
IFPRI: International Food Policy Research Institute
Km: Kilometer
LL: Lowland
ML: Midland
mm: Millimeter
NGOs: Nongovernmental Organizations
RIR: Red Island Red
SNNPR: Southern Nation Nationality of People Region.
